# A case of vasa corona ruptured pseudoaneurysm: case report and review of literature

**DOI:** 10.1007/s00701-025-06531-6

**Published:** 2025-04-22

**Authors:** Hikaru Nakamura, Tsuyoshi Izumo, Kazuaki Okamura, Shota Yoshimura, Nozomi Ueki, Shiro Baba, Kenta Ujifuku, Yoichi Morofuji, Takeshi Hiu, Koichi Yoshida, Takayuki Matsuo

**Affiliations:** 1 Department of Neurosurgery, Nagasaki University Graduate School of Biomedical Sciences, Nagasaki, Japan; 2 Department of Neurosurgery, Nagasaki Prefecture Shimabara Hospital, Nagasaki, Japan; 3 Department of Neurosurgery, Gifu University Graduate School of Medicine, 1 - 1 Yanagido, Gifu, 501 - 1194 Japan; 4 Department of Tumor and Diagnostic Pathology Atomic Bomb Disease Institute, Nagasaki University, Nagasaki, Japan

**Keywords:** Spinal artery, Subarachnoid hemorrhage, Pseudoaneurysm, Vertebral artery, Posterior inferior cerebellar artery

## Abstract

Ruptured aneurysms or pseudoaneurysms associated with the vasa corona, in the absence of cerebral arteriovenous malformation or dural arteriovenous fistula, are extremely rare but should be recognized as a possible cause of subarachnoid hemorrhage (SAH). We report the case of a 51-year-old female who presented with SAH due to a ruptured vasa corona pseudoaneurysm, with no associated anatomical abnormalities involving the vertebral artery or the posterior inferior cerebellar artery. She recovered after direct surgery. In previous cases, anterior spinal artery dilatation due to posterior inferior cerebellar artery anomalies prompted endovascular treatment. A thorough assessment remains crucial, and direct surgery can be effective.

## Introduction

Ruptured aneurysms or pseudoaneurysms associated with the vasa corona, in the absence of cerebral arteriovenous malformations or dural arteriovenous fistulas, are extremely rare [1, 2]. We describe the first case of a ruptured vasa corona pseudoaneurysm, without any other obvious anatomical abnormalities in the vertebral artery (VA) or posterior inferior cerebellar artery (PICA), treated successfully through direct surgery.

## Case report

**Fig. 1:**
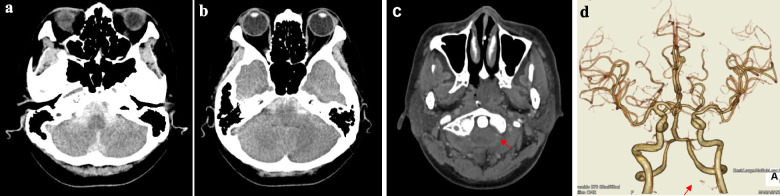
CT scan revealed subarachnoid hemorrhage (SAH) (**a**, **b**). CT angiography (CTA) showed a punctate contrast effect (red arrow) on the left anterior surface of the C1 cervical spinal cord. No stenosis or occlusion of the vertebral artery (VA) or posterior inferior cerebellar artery (PICA) was noted (**c**, **d**)

**Fig. 2:**
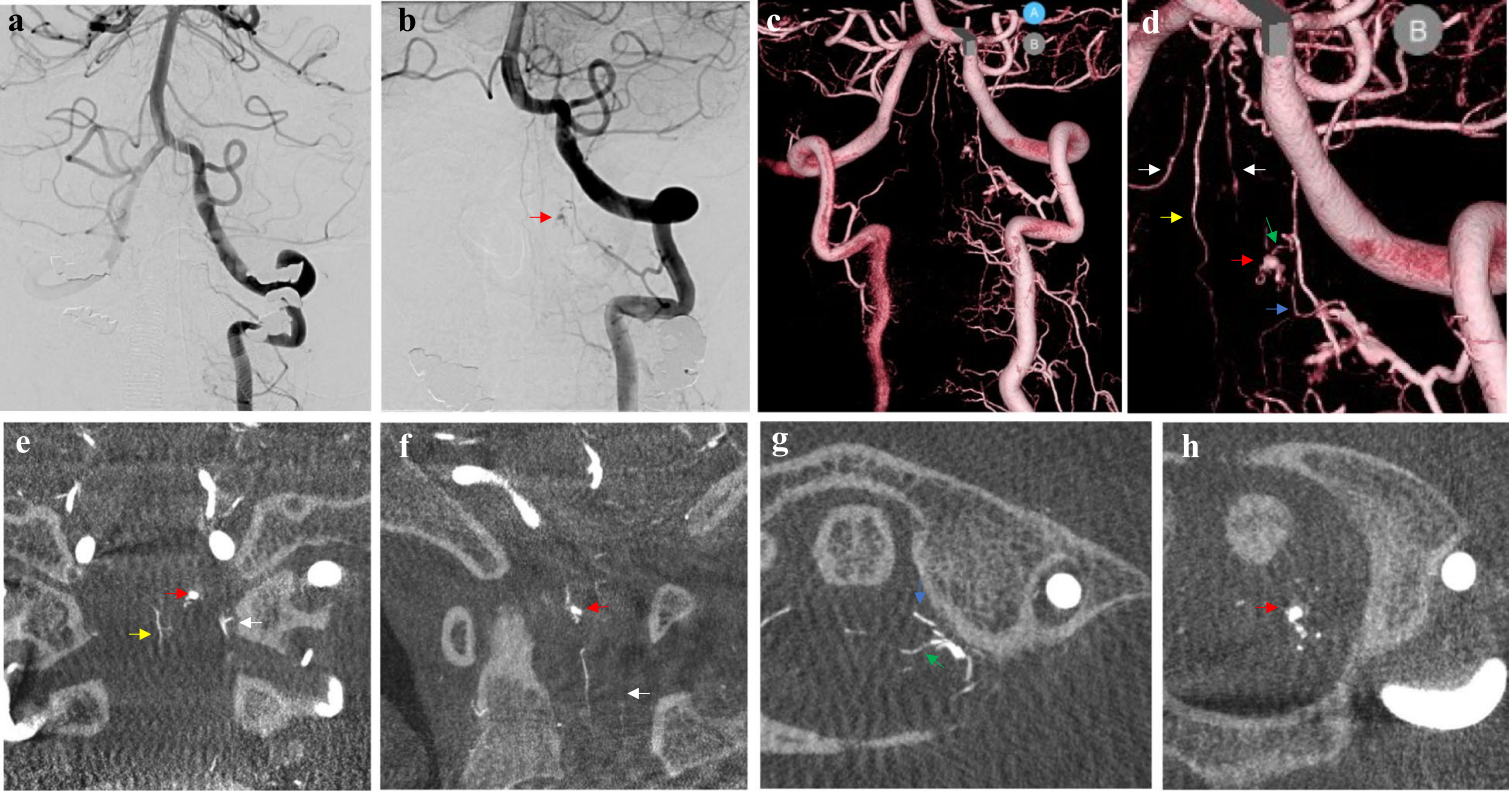
Imaging following left vertebral artery (VA) injection on days 1 (**a**) and 16 (**b**–**d**). Red arrows indicate a microaneurysm-like structure on the anterior surface of the C1 cervical spinal cord. The radiculomedullary (blue arrow) and radiculopial arteries (green arrow), running laterally on the left lateral surface of the anterior spinal artery (ASA) (yellow arrow) and lateral spinal artery (LSA) (white arrow), were identified as mother vessels of this aneurysm using Con-beam CT (**e**–**h**)

A 51-year-old female with no prior medical history presented with headache and vomiting and sought treatment at another hospital. She was diagnosed with subarachnoid hemorrhage (SAH) and subsequently transferred to our hospital. Upon admission, her Glasgow Coma Scale score was 15, and had no neurological deficits. Computed tomography (CT) revealed a subarachnoid hemorrhage extending from the basilar to the medullary cistern (Fig. 1a, b). She was classified as Grade I on both the World Federation of Neurological Surgeons (WFNS) scale and the Hunt and Kosnik scale. CT angiography (CTA), performed on the day of admission, identified a punctuate contrast on the left anterior surface of the C1 cervical spinal cord and was thought vascular abnormalities, such as aneurysms or pseudoaneurysm, might be present. There was no stenosis or occlusion of the VA or PICA, nor were any other obvious anatomical abnormalities observed (Fig. 2c, d).

However, the left VA injection performed the day after admission did not show the lesion that was previously revealed by CTA, suggesting that it could have become thrombosed (Fig. 2a). Therefore, we decided to perform follow-ups while managing the spasms. The left VA injection performed on day 16 revealed a microaneurysm-like structure on the left anterior surface of the C1 cervical spinal cord that had been observed using CTA at the time of admission (Fig. 2b–d). Cone beam CT revealed that the mother vessels were the radiculomedullary and radiculopial arteries. Because the aneurysm was located near the anterior surface of the cervical spinal cord, we judged it to be a vasa corona aneurysm as a preoperative diagnosis (Fig. 2e–h).

**Fig. 3:**
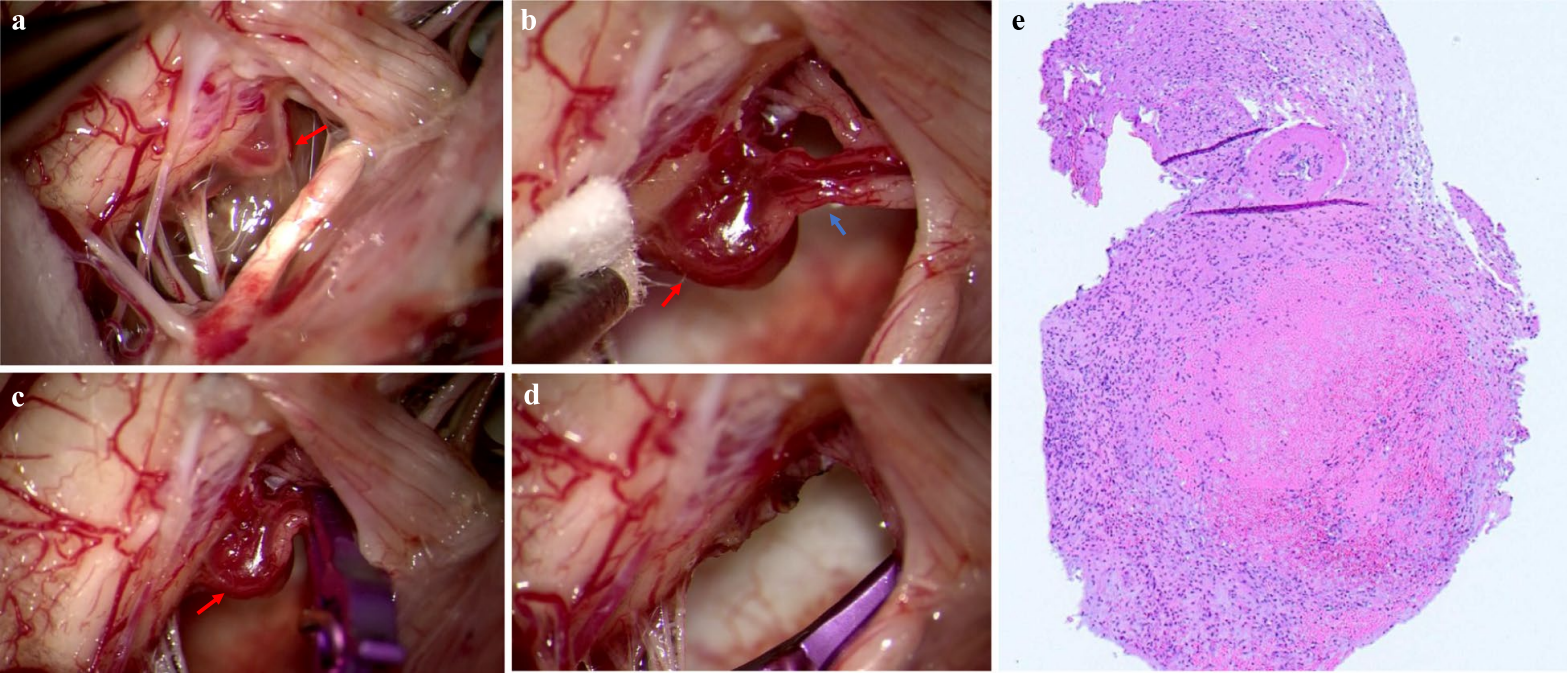
**a**, **b** Identification of a microaneurysm-like structure (red arrow) continuous with the radiculomedullary artery (blue arrow). **c** Localization of clip placement. **d** Coagulation and resection of the radiculomedullary artery and vessels draining from the ventral side. **e** Pathological diagnosis revealed pseudoaneurysm diagnosis

We performed a left suboccipital craniectomy and C1 laminectomy. A microaneurysm-like structure continuous with the radiculomedullary artery was identified (Fig. 3a, b) and clipped (Fig. 3c). These vessels were coagulated and resected along with the vessels draining from the ventral side (Fig. 3d). Pathological diagnosis revealed small muscular vessels, thrombus, granulation tissue formation around the thrombus, and a recanalized thrombus. Based on these findings, it was considered to be a pseudoaneurysm (Fig. 3e).

The patient progressed well and was discharged with a modified Rankin Scale of 0. Informed consent was obtained from the patient for the publication of this case report and the accompanying images.

## Discussion

As shown in Table 1, there have been only two previous reports of vasa corona ruptured aneurysms or pseudoaneurysm without cerebral arteriovenous malformations or dural arteriovenous fistulas, and all previous reports described an anatomical anomaly of the PICA. These anatomical anomalies could not be identified in the present case, and this is the first report of a patient in whom direct surgery was performed.

**Table 1:** Reported cases of vasa corona and lateral spinal artery ruptured aneurysms or pseudoaneurysm without arteriovenous malformation or dural arteriovenous fistula

Case	Author (year)	Age/Sex	Location of the aneurysm	H&H grade	Angiographic findings	Treatment	Pathological diagnosis	Outcome(mRS)
1	Chen et al. (2001) [3]	72/F	LSA	III	Bilateral VA stenosis	Coil embolization	Not done	4
2	Chen et al. (2001) [3]	69/F	LSA	III	Lt. VA stenosis	Resection of the aneurysm	Aneurysm	6
3	Kubota et al. (2006) [4]	59/F	LSA	No data	Rt. PICA occlusion	Resection of the aneurysm	No mention	No mention
4	Kurita et al. (2009) [5]	61/M	LSA	I	No obvious anomaly	Clipping	No mention	0
5	Morigaki et al. (2012) [6]	78/M	LSA	IV	Bilateral VA occlusion	Coil embolization	Not done	4
6	Mizutani et al. (2016) [1]	42/F	Vasa corona	I	Rt. PICA originating from ASA	Coil embolization	Not done	1
7	Germans et al. (2018) [7]	49/M	LSA	II	Rt. PICA occlusion	Clipping	Not done	0
8	Matsumoto et al. (2020) [2]	44/F	Vasa corona	III	Lt. duplication PICA	NBCA embolization	Not done	0
9	Okamoto et al. (2021) [8]	69/F	LSA	II	Lt.VA occlusion	Resection of the aneurysm	No mention	2
10	Papadimitriou K, et al. (2024) [9]	74/F	LSA	III	No obvious anomaly	clipping	No mention	0
11	Song Y, et al. (2024) [10]	55/M	LSA	II	Lt. AICA and PICA stenosis	clipping	No mention	1
12	Song Y, et al. (2024) [10]	64/M	LSA	I	Bilat. VA stenosis	clipping	No mention	0
13	Song Y, et al. (2024) [10]	73/F	LSA	I	No obvious anomaly	No mention	No mention	No mention
14	Jeon YS, et al. (2024) [11]	51/F	LSA	I	Rt. V4 aplasia	Coil embolization	Not done	0
15	Present case (2024)	51/F	Vasa corona	II	No obvious anomaly	Resection of the aneurysm	Pseudoaneurysm	0

*ASA* anterior spinal artery, *F* Female, *H&H grade* Hunt and Hess grade, *LSA* lateral spinal artery *LT* left, *LVA* vertebral artery, *M* Male *mRS* modified Rankin Scale. *NBCA* N-butyl cyanoacrylate, *PICA* posterior inferior cerebellar artery, *Rt* right

Only 12 LSA [3–8] and 3 vasa corona [1, 2] ruptured aneurysms or pseudoaneurysm in the lateral spinal cord have been described. Of these, nine LSA (75%) and two vasa corona (66.7%) ruptured aneurysm cases had VA or PICA abnormality such as stenosis or occlusion. In addition, severe stenosis was noted, and hemodynamic stress associated with these structural abnormalities was reported as the mechanism of aneurysm occurrence [1–4, 6–8, 12]. However, in this case, no underlying anatomical abnormalities or angiographic findings were confirmed, whereas histopathological findings suggest the possibility of a pseudoaneurysm, which is mostly the main etiology in this case, potentially caused by dissection and fusiform dilation (diffuse and global dilation of the vessel).

Pathological diagnosis was performed in two cases, including our case, which is the first case of a ruptured vasa corona pseudoaneurysm, but we think it is necessary to assess more similar cases for definitive conclusions.

Two previously reported cases of ruptured vasa corona aneurysms were managed endovascularly [1, 2]. In both, an ASA dilatation caused by PICA anomaly allowed microcatheter navigation. Conversely, our case is the first treated via direct surgery; no PICA abnormality or ASA dilatation was identified, making endovascular access difficult. Thus, in the absence of dilated spinal arteries, direct surgery may be necessary.

As for limitations, our report is limited by the small sample size of documented cases and a lack of long-term follow-up data.

In conclusion, we described an extremely rare case in which direct surgery was performed to treat a ruptured pseudoaneurysm in the vasa corona. Even in the absence of anatomical abnormalities or abnormal angiographic findings, it is necessary to conduct a detailed examination of the vasa corona aneurysm. Direct surgery may be effective in such cases.

## Data Availability

No datasets were generated or analysed during the current study.
